# Singlet oxygen luminescence as an *in vivo* photodynamic therapy dose metric: validation in normal mouse skin with topical amino-levulinic acid

**DOI:** 10.1038/sj.bjc.6602331

**Published:** 2005-01-18

**Authors:** M J Niedre, C S Yu, M S Patterson, B C Wilson

**Affiliations:** 1 Department of Medical Biophysics, Ontario Cancer Institute/University Health Network Ontario, Canada; 2 University of Toronto, Toronto, Ontario, Canada; 3 Juravinski Cancer Center,Hamilton, Ontario, Canada; 4 McMaster University, Hamilton, Ontario, Canada

**Keywords:** photodynamic therapy, singlet oxygen, luminescence, dosimetry

## Abstract

Although singlet oxygen (^1^O_2_) has long been proposed as the primary reactive oxygen species in photodynamic therapy (PDT), it has only recently been possible to detect it in biological systems by its luminescence at 1270 nm. Having previously demonstrated this *in vitro* and *in vivo*, we showed that cell survival was strongly correlated to the ^1^O_2_ luminescence in cell suspensions over a wide range of treatment parameters. Here, we extend this to test the hypothesis that the photobiological response *in vivo* is also correlated with ^1^O_2_ generation, independent of individual treatment parameters. The normal skin of SKH1-HR hairless mice was sensitised with 20% amino-levulinic acid-induced protoporophyrin IX and exposed to 5, 11, 22 or 50 J cm^−2^ of pulsed 523 nm light at 50 mW cm^−2^, or to 50 J cm^−2^ at 15 or 150 mW cm^−2^. ^1^O_2_ luminescence was measured during treatment and the photodynamic response of the skin was scored daily for 2 weeks after treatment. As observed by other authors, a strong irradiance dependence of the PDT effect was observed. However, in all cases the responses increased with the ^1^O_2_ luminescence, independent of the irradiance, demonstrating for the first time *in vivo* an unequivocal mechanistic link between ^1^O_2_ generation and photobiological response.

Photodynamic therapy (PDT) is an emerging therapy for the treatment of solid tumours and some nonmalignant conditions (Dougherty *et al*, 1998; Stewart *et al*, 1998). The therapy involves the activation of light-sensitive drugs with a laser or other light source to generate reactive oxygen species (ROS). For most clinically used photosensitisers, the most important ROS is believed to be singlet oxygen (^1^O_2_ (^1^Δ_g_)) (Weishaupt *et al*, 1976). The action of ^1^O_2_ results in modification or destruction of the target tissue and subsequent clinical effects (Schweitzer and Schmidt, 2003).

Since PDT involves three interdependent and dynamic treatment factors (i.e. light, photosensitiser and oxygen), complete and accurate dosimetry is a difficult problem and is the focus of ongoing research by several groups. Several techniques have been proposed (Wilson *et al*, 1997a), such as ‘explicit dosimetry’, in which the quantities of light, drug and oxygen are continuously monitored during treatment. Alternatively, ‘implicit dosimetry’ utilises a surrogate for biological damage, such as the photodegradation of the photosensitiser (fluorescence) during treatment to predict treatment outcome (Wilson *et al*, 1997a; Dysart *et al*, 2002). The focus of the present work is ‘direct dosimetry’, which entails direct measurement of ^1^O_2_ during treatment.

In PDT, ^1^O_2_ is generated by the following type-II pathway (Patterson *et al*, 1990): 
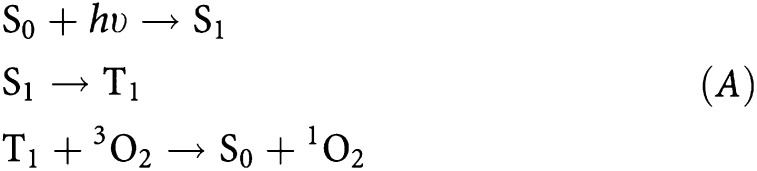
 where S_0_, S_1_ and T_1_ are the ground singlet, first excited singlet and first excited triplet states of the photosensitiser, respectively and ^3^O_2_ and ^1^O_2_ are the ground triplet and first excited singlet states of molecular oxygen, respectively. Once generated, ^1^O_2_ may undergo radiative decay at 1270 nm with a low probability. This luminescence is routinely measurable in solution (Krasnovsky, 1998), but *in vitro* and *in vivo* the lifetime of ^1^O_2_ drops dramatically, from approximately 3 *μ*s to around 100 ns (Moan and Berg, 1991; Schweitzer and Schmidt, 2003) because of the rapid reaction of ^1^O_2_ with surrounding biomolecules. Likewise, the probability of radiative decay drops, such that measurement of this luminescence in biological media has traditionally not been feasible due to limited detector sensitivity and/or temporal response. Nevertheless, there has been significant interest in doing this, both for basic photobiological research and as a potential PDT dosimetry tool (Parker, 1987; Gorman and Rodgers, 1992).

In 2002, we showed for the first time that this is now possible using a novel near-infrared (NIR)-sensitive photomultiplier tube (PMT) (Niedre *et al*, 2002b). Specifically, we measured ^1^O_2_ luminescence *in vitro* from leukaemia cells and *in vivo* in normal liver and skin of Wistar rats, sensitised with aluminium tetrasulphonated phthalocyanine (AlS_4_Pc). Subsequently, Hirano *et al* (2002) also showed that this was possible *in vivo* in implanted murine tumours sensitised with ATX-S10 using the same PMT.

More recently, we published a set of experiments that showed that ^1^O_2_ luminescence was a useful PDT dose metric *in vitro* (Niedre *et al*, 2002a, 2003). Specifically, this demonstrated that the killing of OCI-AML5 leukaemia cells treated with aminolevulinic acid (ALA)-induced protoporphyrin IX (PpIX) PDT correlated very strongly with the ^1^O_2_ luminescence measured during treatment, regardless of initial photosensitiser concentration, irradiance or molecular oxygen concentration. These experiments differed from our initial feasibility study in that the light and photosensitiser conditions were typical of clinical PDT treatments (as opposed to being optimised for generation of ^1^O_2_) and, hence, substantial technical upgrades were required to the detection system.

Following this encouraging first step, we report here a set of experiments that extend this concept to an *in vivo* model, specifically the normal skin of hairless mice sensitised with ALA-PpIX. For this, we used an available green (523 nm) light source, for which the ^1^O_2_ generation and PDT effect were both confined to relatively superficial tissue. This model is based on studies conducted by Robinson *et al* (1998) that attempted to correlate PpIX photobleaching with the observed skin response measured daily for 2 weeks following treatment. Here, we observed (as did Robinson *et al*) a strong irradiance dependence of the response, despite identical ALA concentrations and total treatment fluence. We show that the ^1^O_2_ luminescence generated during treatment correlates well with the observed skin response in all cases, regardless of the treatment fluence or irradiance. To our knowledge, this is the first time that such a correlation has been demonstrated *in vivo.*

As will be discussed, these data support the hypothesis that ^1^O_2_ is the primary ROS involved in PDT *in vivo*. They are also encouraging for the development of ^1^O_2_ luminescence-based preclinical and/or clinical PDT dosimetry systems.

## MATERIALS AND METHODS

### Theory

As described in detail previously (Niedre *et al*, 2002b), the local ^1^O_2_ concentration as a function of time generated by a short laser pulse is given by: 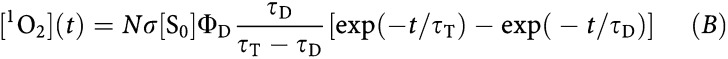 where *N* is the number of photons per cm^2^ in the excitation pulse incident on the sample, σ is the photosensitiser ground state absorption cross-section (cm^2^), [S_0_] is the concentration of the photosensitiser ground state, Φ_D_ is the quantum yield of ^1^O_2_, and *τ*_T_ and *τ*_D_ are the photosensitiser triplet-state lifetime and ^1^O_2_ lifetime, respectively.

The total number of photons emitted in the radiative decay of ^1^O_2_ at 1270 nm is given by 
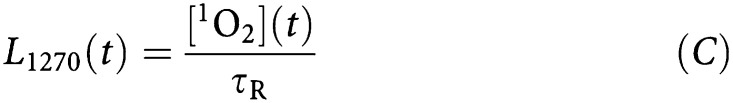
 where *τ*_R_ is the radiative lifetime of ^1^O_2_ in the specific environment. Equation (C) can be integrated over time to give the total number of photons emitted after excitation by a single laser pulse as 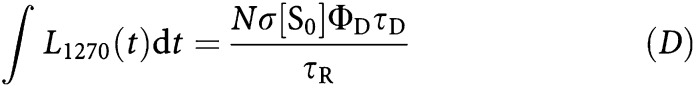 Hence, the concentration of ^1^O_2_ generated in a sample is directly proportional to the total emitted luminescence.

We approximate integral (D) experimentally by counting the total luminescence in the interval between 2 and 90 *μ*s following the laser pulse and subtracting background contributions. Since the laser was operating at 10 kHz, by counting in this interval the system was actively measuring 88% of the time. As in our earlier work, the contributions from the first 2 *μ*s were rejected due to strong fluorescence contributions to the signal from the photosensitiser and some optical elements. Since the kinetics of the ^1^O_2_ luminescence were determined by Equation (B), we could expect that, despite the extremely short *τ*_D_ in tissue, the ^1^O_2_ full-time curve would last several *τ*_T_. Our previous estimates for *τ*_T_ in tissue were between 25 and 30 *μ*s (Niedre *et al*, 2002b), and in the present experiments were confirmed to be 30 to 40 *μ*s (data not shown). However, even if *τ*_T_ was very long (e.g. due to very low pO_2_) the loss of counts would be small (i.e. <12%) due to the high duty cycle of the system.

### Apparatus

**Figure 1:**
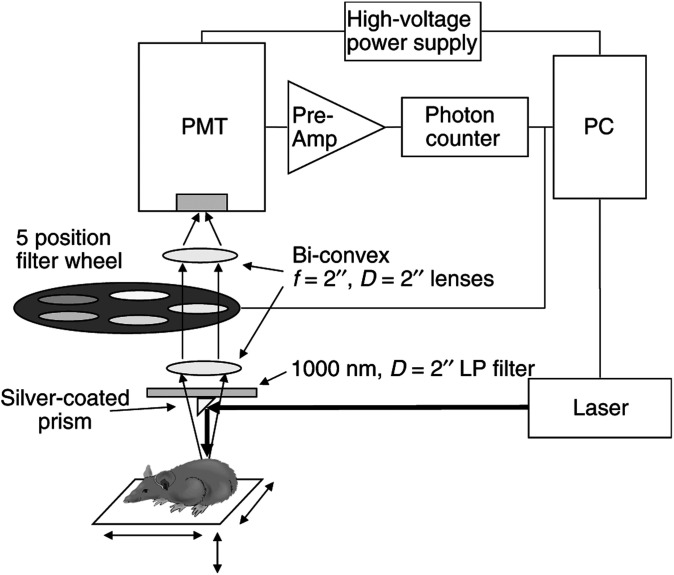
Schematic of the experimental system.

The apparatus used to measure ^1^O_2_ luminescence *in vivo* is shown in Figure 1. This system has been described in detail elsewhere (Niedre *et al*, 2003), with modifications made for the current experiments as follows: (i) The NIR PMT (R5509-14, Hamamatsu Corp., Bridgewater, NJ, USA) was mounted vertically, so that the detector and collection optics were above the animals. As before, the operating voltage of the PMT was set to −1500 V using a high voltage power supply (model SR445, Stanford Research Systems, Sunnyvale, CA, USA); (ii) a small, 4 mm × 4 mm silver-coated prism (01-PRS-411, Melles Griot Inc., Nepean, ON, Canada) was mounted directly in front of the silicon long-pass filter and used to redirect the laser beam 90° towards the skin surface. This allowed illumination of the skin while maintaining close positioning between it and the detection optics and to give a high numerical aperture for maximum light collection, and (iii) the multichannel scalar was replaced with a high-speed multiscalar module (Becker and Hickl MSA-300, Boston Electronics, Brookline, MA, USA), which allowed us to operate the laser at 10 kHz without loss of signal due to speed limitations of the electronics.

As with our earlier studies (Niedre *et al*, 2002a, 2003), the laser was a 523 nm diode-pumped, Q-switched frequency-doubled Nd : YLF (QG-523-500; Crystalaser Inc., Reno, NV, USA) with a pulse width of ∼10 ns. The irradiance at the skin was controlled using a set of neutral density filters (FW2AND, Thor Labs Inc., Newton, NJ, USA). As also noted in our earlier studies (Niedre *et al*, 2002b, 2003), there are other potential sources of luminescence in the NIR range besides ^1^O_2_ luminescence, including detector dark counts, luminescence from optical components, tissue auto-fluorescence, and photosensitiser fluorescence and phosphorescence. Spectral discrimination of the detected light was, therefore, achieved using a set of five narrow-band filters centred at 1212, 1240, 1272, 1304 and 1332 nm (OD3 blocking, 20 nm FWHM; Omega Optical, Brattleboro, VT, USA) mounted on a motorised filter wheel in front of the detector. For simplicity, these will be referred to as the 1210, 1240, 1270, 1300 and 1330 nm filters. The system was automated with a personal computer so that the filter sequence could be customised between experiments.

The mice were placed on a translatable X–Y–Z platform and the skin was kept flat with small clamping arms during treatment. A pair of small 1 mW, 635 nm lasers (CPS180; Thor Labs) was aligned so as to intersect at the focal point of the collection optics. This ensured that the treated spots were positioned so that the illumination/collection geometry was consistent between experiments. These lasers were shut off immediately after positioning the animal and were on for only a few seconds.

### Photodynamic therapy

A total of 39 female hairless mice were used (SKH1-HR, Charles River Laboratories Inc., Wilmington, MA, USA), 7–12 weeks old. These were maintained on a low-fluorescence chow diet for at least 2 weeks prior to treatment. They were sensitised 4 h prior to irradiation with a distilled water solution of 20% ALA (Sigma Chemical Co., St Louis, MO, USA) with 2 M NaOH (Sigma) added to raise the pH to 4 and 5% carboxymethyl cellulose (Sigma) added to increase the viscosity. This solution was applied topically to two spots (each ∼2 × 2 cm^2^) on the dorsal skin and covered with a transparent adhesive dressing (Tegaderm 1626W, 3M Health Care, St Paul, MN, USA). Two PDT treatments were performed on each mouse in order to minimise the total number of animals used in the study. These treatments were chosen randomly from the set described below, so that the left side and right side generally received different treatments. Immediately before the application of ALA, the stratum corneum was stripped using medical tape to facilitate diffusion of the ALA into the epidermis.

Since we had not shown previously that it was possible to measure ^1^O_2_ luminescence *in vivo* specifically with ALA-PpIX, a pilot study was conducted on a group of nine animals. The first three were sensitised with ALA-PpIX as above, and irradiated with 50 mW cm^−2^ treatment light, a second set of three were unsensitised controls and the final three were sensitised with ALA-PpIX and euthanised by intracardiac injection of T-61 (Houchst Roussel Vet, Whitby, SK, Canada) 5 min prior to irradiation. The last set allowed us to check the effect of hypoxia on the ^1^O_2_ signal.

After this initial investigation, full treatments were performed to investigate the relationship between ^1^O_2_ luminescence and PDT treatment response. The treatments were repeated six times in all cases and, as summarised in Table 1, were as follows: (i) a irradiance of either 15, 50 or 150 mW cm^−2^ was delivered to a constant total fluence of 50 J cm^−2^; (ii) a total fluence of either 5.5, 11, or 22 J cm^−2^ was delivered at a constant irradiance of 50 mW cm^−2^; (iii) unsensitised control animals were irradiated with 50 J cm^−2^ at either 15, 50 or 150 mW cm^−2^ and; (iv) control animals were sensitised but not irradiated. In cases (i–iii) the irradiated spot was 7 × 7 mm^2^. Since the experiments were performed over a period of several months, the order in which they were performed was randomised in order to minimise any bias, for example in the system sensitivity. For practical reasons, these were not exactly the same treatments performed by Robinson *et al* (1998), but are all in the same range of light and drug parameters used.

### Skin scoring

Following treatment, the irradiated spot was marked and the animals were housed in darkness for 24 h and then observed daily for 2 weeks. On each day, an observer, blinded to the PDT treatment, assigned a numbered skin response score for each treated spot in the range 0–6, as summarised in Table 2. Photographs of the treated spots were taken with a calibrated colour card each day to document the response. All experiments were performed in compliance with the guidelines of the Ontario Cancer Institute Animal Care Committee.

### Singlet oxygen measurement and data analysis

The detection wavelength was selected by positioning the appropriate band-pass filter in front of the PMT. In these experiments, the system was set up to acquire for 345 000 laser pulses per filter. This took 37 s, including filter wheel motion and data processing.

Near-infrared luminescence measurements were made during all of the above experiments, including unsensitised controls. All signals were corrected for minor differences in the transmission characteristics of the optics at each wavelength. For the pilot group, measurements were made at all five wavelengths, giving more spectral information. For the animals in which full treatments were given, only three wavelengths (1240, 1270 and 1300 nm) were used in order to reduce the acquisition time, which totaled 111 s.

The measured spectra in the sensitised animals were corrected for background contributions by subtracting the mean spectrum from the control animals irradiated at the same irradiance. The ^1^O_2_ luminescence signal was then calculated as the photon counts at 1270 nm minus the average of the counts at 1240 and 1300 nm (i.e. the linearly interpolated background at 1270 nm). The resulting value was then multiplied by 3, since the system acquired at each wavelength only 1/3 of the time.

## RESULTS

**Figure 2:**
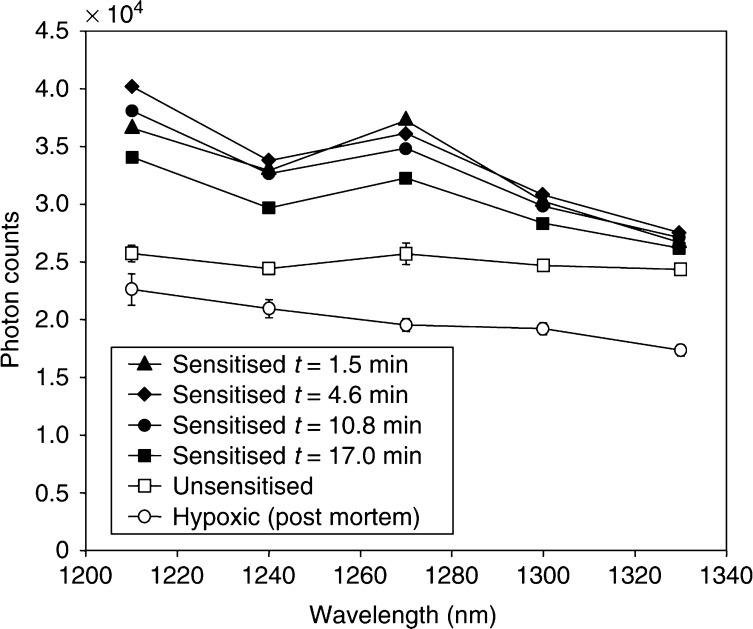
Typical NIR spectra measured from single sensitised, unsensitised and hypoxic animals. For the sensitised animal, the individual spectra were measured at different times during treatment. For the control and hypoxic animals, error bars reflect the standard deviation of four spectra acquired for the same animal.

Figure 2 shows typical, 5-wavelength spectra measured in the pilot group, including the sensitised, unsensitised and euthanised animals (50 mW cm^−2^). As expected for ^1^O_2_ luminescence, a clear 1270 nm peak was observed for the live sensitised animals. As will be demonstrated below, significant variability was observed both in terms of ^1^O_2_ generation and treatment response, even between animals receiving PDT under the same conditions. For this reason, representative data from a single sensitised animal are shown, as opposed to the average of the ^1^O_2_ luminescence observed from all of the live sensitised animals. A small but still significant peak at 1270 nm was observed in the unsensitised control animals, most likely due to ^1^O_2_ generated from naturally occurring porphyrins in the skin. No peak was observed at 1270 nm in the sensitised but euthanised animals, confirming the oxygen dependence of the signal. Hence, the system was capable of measuring ^1^O_2_ luminescence in this *in vivo* model.

**Figure 3:**
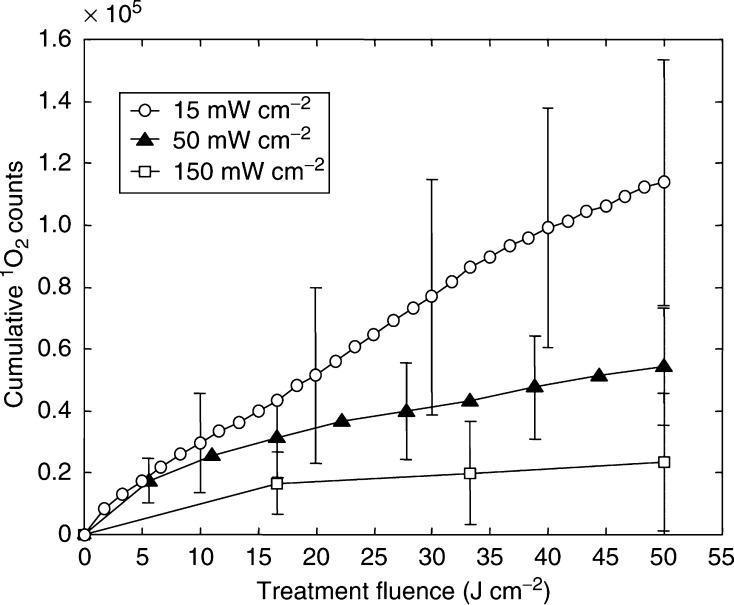
Cumulative ^1^O_2_ luminescence in sensitised animals irradiated to 50 J cm^−2^ at 15, 50 or 150 mW cm^−2^. Each point represents the mean for six animals, ±1s.d. The lines are simply to guide the eye and typical error bars are shown.

Figure 3 shows the average, cumulative ^1^O_2_ luminescence for sensitised animals treated with 15, 50 and 150 mW cm^−2^ up to a total fluence of 50 J cm^−2^, after subtracting the mean control (unsensitised) spectra at the same irradiances. The total ^1^O_2_ luminescence decreased with increasing irradiance. This trend was statistically significant between the three groups: specifically, one-tailed Student's *t*-tests yielded *P*=0.005 comparing the 15 and 50 mW cm^−2^ treatments and *P*=0.014 between the 50 and 150 mW cm^−2^ treatments. Note that the error bars in Figure 3 represent the standard deviation between the six animals in each experiment. The error due to photon counting statistics was negligible compared to this systematic variability. The relevance of this observation is discussed below.

**Figure 4:**
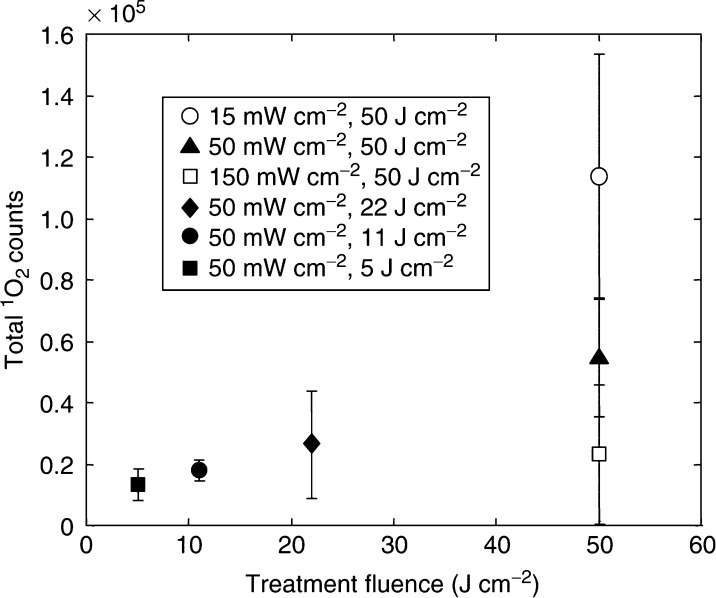
Total ^1^O_2_ luminescence as a function of total fluence for all treatment groups (means±1s.d. in six animals).

Similar curves were measured for all other treatment groups. Figure 4 summarises the mean total ^1^O_2_ luminescence observed in each group. As would be expected, the total ^1^O_2_ luminescence increased with radiant exposure (fluence) at a constant irradiance.

**Figure 5:**
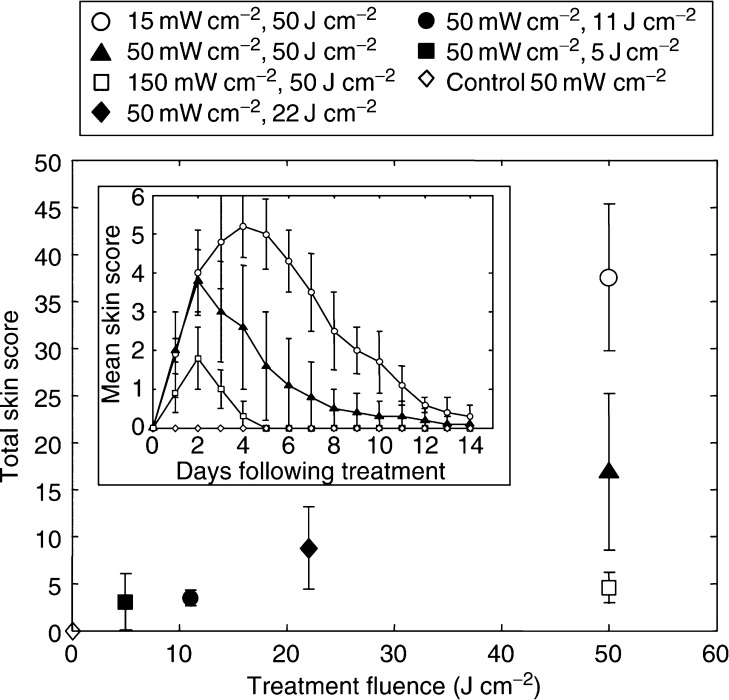
Total skin score (means±1s.d. in six animals) as a function of total fluence for all treatment conditions. Inset: skin score as a function of time following treatment in days (means±1s.d. in six animals).

Figure 5 (inset) shows the skin scores for spots treated with 50 J cm^−2^ at varying irradiances, corresponding to the treatments in Figure 3, as well as the unsensitised controls at 50 mW cm^−2^. The unsensitised animals had no observable response to light alone at 15 or 150 mW cm^−2^ or to ALA alone (data not shown for brevity). For the sensitised animals, the skin response increased significantly with decreasing irradiance. This was statistically significant: *P*=0.001 between 15 and 50 mW cm^−2^ and *P=*0.003 between 50 and 150 mW cm^−2^. This irradiance dependence was also observed by Robinson *et al* (1998), and similar effects have been reported by other authors in different *in vitro* and *in vivo* models (Feins *et al*, 1990; Gibson *et al*, 1990; Foster *et al*, 1993; Sitnik *et al*, 1998), and has usually been attributed to photochemical depletion of oxygen at high irradiances. The total skin scores as a function of fluence for all treatments are summarised in Figure 5. These were defined as the sum of the 14 individual daily scores over the 2-week period in each case. Again, these are generally consistent with those obtained by Robinson *et al* (1998).

A striking feature in Figures 3, 4 and 5 is the relatively large variability in the measurements for nominally identical PDT treatments. As will be discussed, this variability was primarily due to animal-to-animal differences in ALA uptake and/or PpIX synthesis, local skin *p*O_2_ and relative photosensitivity of the skin, as opposed to true ‘experimental error’. The variability observed here was not atypically large, but serves to illustrate the difficulty inherent in predicting the outcome of PDT treatments based on administered light and photosensitiser dose alone.

**Figure 6:**
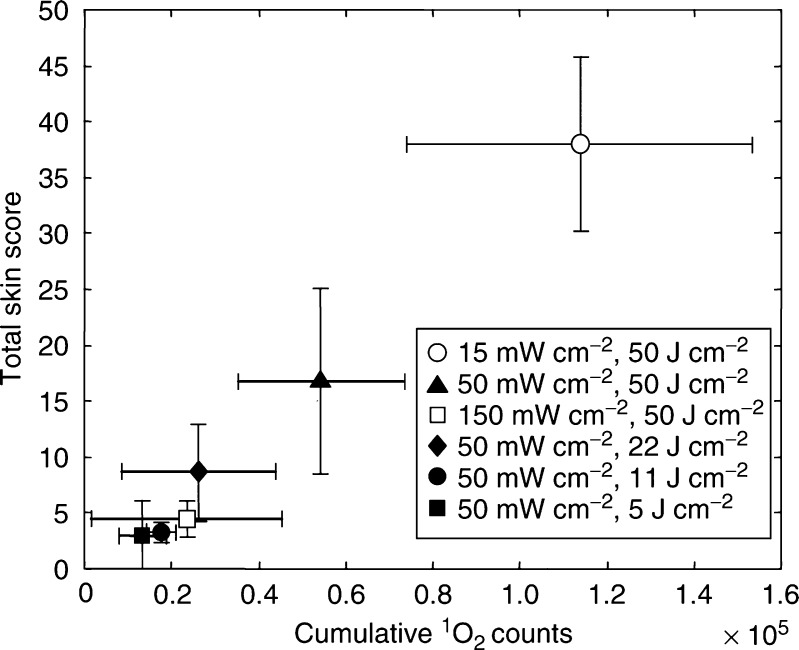
Total skin score (means±1s.d.) as a function of total ^1^O_2_ luminescence (means±1s.d.).

**Figure 7:**
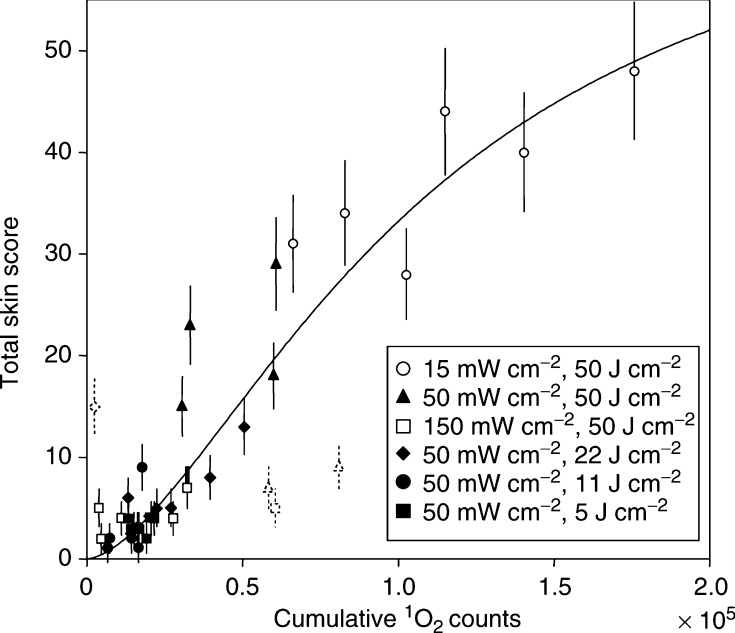
Total skin score as a function of total ^1^O_2_ luminescence for all individual data points that comprise the figure. The curve is the fit to Equation (E) after removal of the four outliers (open points). The error bars indicate the assumed systematic uncertainty in the visual scoring, taken as ±0.5 units on each score.

The total skin score as a function of the total ^1^O_2_ luminescence for all treatments is shown in Figure 6. There is a strong correlation with the ^1^O_2_ luminescence, regardless of the fluence or irradiance used. Figure 7 shows the individual data points that comprise Figure 6, together with the best *χ*^2^ fit to a three-parameter sigmoidal curve (constrained to pass through the origin) of the form: 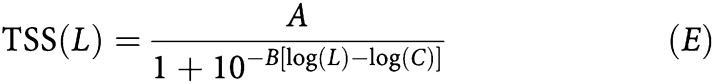 where TSS is the total skin score and *L* is the total ^1^O_2_ luminescence measured. The use of this functional form for the response has no *a priori* mechanistic basis at this time, but is a convenient way to summarise the data. This fit was performed with four outliers removed (dotted symbols in Figure 7) and this fit yielded *A*=71, *B*=1.6 and *C*=108 000 and a reduced *χ*^2^ of 2.0 (*χ*^2^/NDF=5.1 with all data points included).

## DISCUSSION

### Technical issues

As with our previous work (Patterson *et al*, 1990; Niedre *et al*, 2002a, 2002b; Niedre *et al*, 2003), we chose to use a set of NIR band-pass filters for spectral discrimination of the detected light rather than a monochromator (Hirano *et al*, 2002), since it allowed for maximum optical throughput and minimised the distance between the source and detector. Furthermore, after we verified that our system was capable of measuring ^1^O_2_ luminescence in this *in vivo* model, we were able to use a set of only three filters, since the 1270 nm peak was unambiguous.

The addition of a small, 4 × 4 mm prism in front of the collection optics allowed close positioning of the animals while allowing irradiation of the treatment spot. Since less than 1% of the field of view of the detector was blocked by this prism, the impact on signal collection was minimal.

Accurate positioning of the skin spot during ^1^O_2_ luminescence measurements was important, since different animals were measured over an extended period of time and absolute ^1^O_2_ luminescence measurements were compared. The pair of alignment lasers ensured that the animals were positioned reproducibly with an accuracy (height) of about ±0.5 mm at the focal plane of the detection system, so that the complete system response was consistent. Furthermore, the laser irradiance, measured before each treatment was stable to ±5% RMS, while the power supply for the PMT was stable to ±1 V.

### Singlet oxygen as an *in vivo* dose metric

The key finding in this study is that treatment response in normal mouse skin *in vivo* correlates strongly ^1^O_2_ luminescence measured during PDT. Furthermore, this was the case even for a range of radiant exposures and irradiances over which the response showed significant variation. In addition, as discussed earlier, significant variability was observed in both ^1^O_2_ luminescence and skin response for nominally identical treatments. The fact that the points from the individual experiments follow a single (sigmoidal) curve (Figure 7) illustrates the ability of this technique to account for variability in treatment factors such as PpIX concentration and skin *p*O_2_. Although significantly more scatter (and outlying data points) from the parent curve is evident in Figure 7 than in our analogous *in vitro* studies (Niedre *et al*, 2003), this was not unexpected, since more variability should exist in the photobiological response between individual mice than between sets of cells from clonal populations. Also, the skin scoring system used in these experiments is somewhat subjective and may have contributed to this scatter.

The fraction of ^1^O_2_ that undergoes radiative decay relative to all ^1^O_2_ generated in the treatment volume (regardless of de-excitation pathway) is given by the ratio of the ^1^O_2_ lifetime to the luminescence lifetime, that is, *τ*_D_/*τ*_L_ (Lamola, 1971). Hence, although the probability of radiative decay is extremely low *in vivo* (Niedre *et al*, 2002b), the emitted luminescence is tightly coupled to the ‘active’, nonradiating fraction of ^1^O_2_. The fact that the ^1^O_2_ luminescence measured here correlates extremely well with the treatment response further demonstrates this relationship. In addition, the ^1^O_2_ luminescence signal was undoubtedly heterogeneous in origin in that, for example, the photosensitiser was likely concentrated in specific tissue and/or cellular compartments (e.g. mitochondrial membrane; Wilson *et al*, 1997b) and the oxygenation is higher in membranes than in cytosol. However, the proportion of radiative *vs* nonradiative ^1^O_2_ was consistent between experiments, since we used the same photosensitiser and application conditions in all cases. This might be a complicating factor if, for example, ^1^O_2_ luminescence was compared to treatment response using different photosensitisers and administration conditions. Moreover, the effect of tissue optics in these experiments was minimal, since we deliberately chose a relatively homogeneous tissue model as opposed to, for example, a murine tumour model (Robinson *et al*, 1998).

This work, together along with our earlier dose–response studies in cells, supports the generally-held hypothesis that ^1^O_2_ is the important cytotoxin involved in PDT. Furthermore, since the relationship between ^1^O_2_ generation and treatment response holds *in vivo*, this work further demonstrates the utility of using ^1^O_2_ luminescence as a PDT dosimetry tool. However, the possibility that ^1^O_2_ is only one of the ROS generated cannot be discounted, nor can the possibility that other ROS are more important for different photosensitisers.

### Irradiance effects

**Figure 8:**
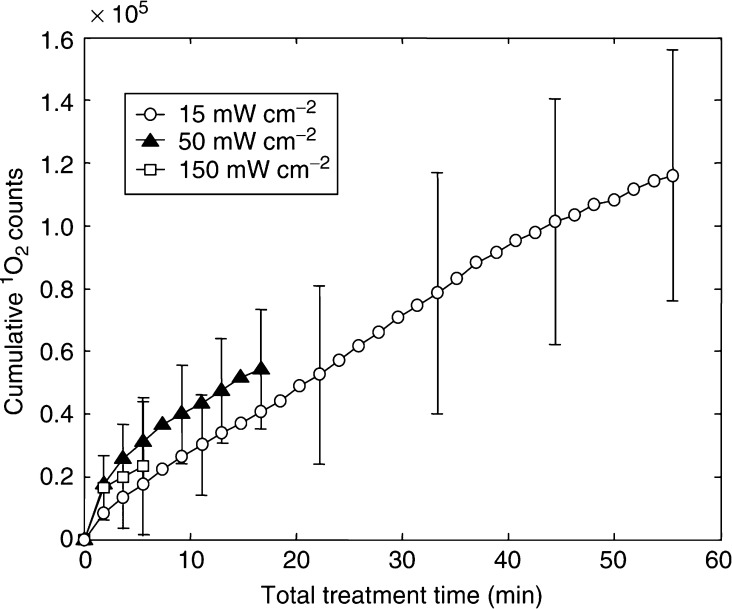
Total ^1^O_2_ luminescence *vs* total treatment time for animals that received 50 J cm^−2^ at 15, 50 or 150 mW cm^−2^ (means±1s.d.).

Strong irradiance effects were observed in these experiments, both in terms of ^1^O_2_ luminescence generation and treatment response. Irradiance effects have been observed by other authors (Feins *et al*, 1990; Gibson *et al*, 1990; Foster *et al*, 1993; Robinson *et al*, 1998; Sitnik *et al*, 1998) and have usually been attributed to rapid photochemical depletion of molecular oxygen at higher irradiances. The ^1^O_2_ luminescence measurements here appear to be consistent with this interpretation. Specifically, Figure 8 shows the cumulative ^1^O_2_ luminescence (as in Figure 3) as a function of total *treatment time*. The fact that all of the curves appear to reach approximately the same terminal slope regardless of irradiance implies that ^1^O_2_ generation was not limited by the number of excitation photons in each laser pulse, but rather by the available molecular oxygen and/or photosensitiser ground state. We are currently investigating both of these possibilities in more detail.

### Other observations

Robinson *et al* (1998) observed rapid photobleaching of the photosensitiser: for example, greater than 90% of the PpIX was bleached after 10 J cm^−2^ at their lowest irradiance. This appears to contradict our data, since the incremental ^1^O_2_ generation at 15 mW cm^−2^ appeared unaffected by the bleaching of the photosensitiser and decreased only slightly at later time points (Figure 8). A possible explanation for this is the formation of photosensitive photoproducts during PpIX irradiation (Finlay *et al*, 2001). These photoproducts are known to absorb at 523 nm and therefore may have acted as secondary photosensitisers, allowing sustained photodynamic generation of ^1^O_2_. Alternatively, it is possible that sufficient PpIX was always present during treatment, so that the concentration of molecular oxygen was the limiting factor in ^1^O_2_ generation even after several orders of magnitude of photosensitiser photobleaching. This further illustrates the potential value of ^1^O_2_ luminescence-based dosimetry, since complicating factors such as photobleaching, formation of photosensitive photoproducts and tissue oxygenation are all implicitly incorporated into a single measurement.

Analysis of the data of Figure 7 showed that the lowest ^1^O_2_ luminescence counts measured from any treatment that resulted in an individual skin response score of 5 or higher (i.e. scab formation/necrosis) at any day post-treatment was 61 000 photon counts. Given the system geometric collection efficiency (0.02), quantum efficiency of the detector (0.01), optical throughput of the collection optics (0.2) and the probability of radiative decay of ^1^O_2_ in tissue (=*τ*_D_/*τ*_L_∼100 ns/5.55 s ∼2 × 10^−8^) (Niedre *et al*, 2002b), this was equivalent to ∼7 × 10^16^ molecules of ^1^O_2_ in the treatment volume. Assuming a typical cell density in tissue of around 10^9 ^cm^−3^ and, given that the effective treatment volume was approximately 0.5 cm^2^ with a depth of 25 *μ*m (Robinson *et al*, 1998), the ^1^O_2_ necrosis threshold for this model was about 5.8 × 10^10^ molecules of ^1^O_2_ per cell. This is can be compared to Georgakoudi *et al*'s (1997) estimate of ∼7 × 10^9^ molecules of ^1^O_2_ per cell in EMT6 spheroids treated with Photofrin-PDT, and Farrel *et al*'s (1991) estimate of ∼5 × 10^8^ per cell in rat liver treated with Photofrin-PDT, Interestingly, it is also significantly higher than our own estimate of the 5.6 × 10^7^ molecules ^1^O_2_ per cell to achieve 1/e cell death in OCI-AML5 cells *in vitro* with ALA-PpIX (Niedre *et al*, 2003). This apparently higher threshold for the skin response likely reflects the relative insensitivity of normal mouse epidermal cells *vs* leukaemia cells, and the lack of a vascular component in the treatment response to ALA-PpIX PDT *vs* that of Photofrin-PDT. It should also be noted that this result is sensitive to the assumed treatment depth; here, we have assumed that PpIX synthesis is confined to the epidermis as described by Kennedy and Pottier (1992) and Robinson *et al* (1998), but there may have been contributions from the dermis as well. This would lead to the above threshold value being overestimated.

## CONCLUSIONS

In summary, this work demonstrates the potential value of ^1^O_2_ luminescence as a dose metric *in vivo.* Combined with our previous studies, the prospect of further extending this work towards a clinical dosimetry system is encouraging. We plan to repeat these studies next in an implanted tumour model that has an increased level of complexity due to inherent heterogeneity in optical properties, photosensitiser uptake and oxygenation. This may require further modification of the system to provide spatial information as well as single, volume-averaged ^1^O_2_ luminescence measurements. If successful, we then plan to perform a clinical demonstration of ^1^O_2_ luminescence measurements during PDT, probably initially on skin cancer patients.

## Figures and Tables

**Table 1:** Summary of the PDT treatments, repeated 6 times in all cases

**Fluence rate (mW cm^−2^)**	**Fluence (J cm^−2^)**	**Treatment time (s)**	**ALA concentration**
150	50	333	20%
50	50	1000	20%
15	50	3333	20%
50	22	440	20%
50	11	220	20%
50	5.5	110	20%
15, 50, 150	50	333, 1000, 3333	0%
0	0	0	20%

**Table 2:** Visual skin-response scoring system (Robinson *et al*, 1998)

**Score**	**Observation**
0	No observable effect
1	Mild erythema
2	Moderate erythema
3	Strong erythema
4	Dry desquamation
5	Thin scab formation
6	Thick scab formation
